# Artificial Intelligence Applied to Flavonoid Data in Food Matrices

**DOI:** 10.3390/foods8110573

**Published:** 2019-11-14

**Authors:** Estela Guardado Yordi, Raúl Koelig, Maria J. Matos, Amaury Pérez Martínez, Yailé Caballero, Lourdes Santana, Manuel Pérez Quintana, Enrique Molina, Eugenio Uriarte

**Affiliations:** 1 Facultad de Ciencias Aplicadas, Universidad de Camagüey Ignacio Agramonte Loynaz, Cincunvalación Norte km 5 1/2, 74650 Camagüey, Cuba; 2 Facultad de Farmacia, Campus vida, Universidad de Santiago de Compostela, 15782 Santiago de Compostela, Spain; 3 CIQUP/Department of Chemistry and Biochemistry, Faculty of Sciences, University of Porto, 4169-007 Porto, Portugal; 4 Facultad de Ciencias de la Tierra, Universidad Estatal Amazónica, km 2 ½ vía Puyo a Tena (Paso Lateral), Puyo 032892-118, Ecuador; 5 Instituto de Ciencias Químicas Aplicadas, Universidad Autónoma de Chile, Santiago 7500912, Chile

**Keywords:** flavonoid, artificial intelligence, total antioxidant capacity

## Abstract

Increasing interest in constituents and dietary supplements has created the need for more efficient use of this information in nutrition-related fields. The present work aims to obtain optimal models to predict the total antioxidant properties of food matrices, using available information on the amount and class of flavonoids present in vegetables. A new dataset using databases that collect the flavonoid content of selected foods has been created. Structural information was obtained using a structural-topological approach called TOPological Sub-Structural Molecular (TOPSMODE). Different artificial intelligence algorithms were applied, including Machine Learning (ML) methods. The study allowed us to demonstrate the effectiveness of the models using structural-topological characteristics of dietary flavonoids. The proposed models can be considered, without overfitting, effective in predicting new values of Oxygen Radical Absorption capacity (ORAC), except in the Multi-Layer Perceptron (MLP) algorithm. The best optimal model was obtained by the Random Forest (RF) algorithm. The *in silico* methodology we developed allows us to confirm the effectiveness of the obtained models, by introducing the new structural-topological attributes, as well as selecting those that most influence the class variable.

## 1. Introduction

The relationship between dietary intake of bioactive antioxidants and health needs new approaches and studies for a better understanding. Research in this field is limited by the high number of bioactive compounds, which also hinders the development of analytical techniques and the availability of benchmarks [1]. Studying the currently growing and dispersed information on dietary phytochemicals is a huge challenge [2].

Several food databases were prepared based on the emerging Food Composition Database (FCDB) [3,4]. These databases focus on the composition of bioactive substances, including flavonoids and other polyphenols.

The flavonoid FCDB provides researchers with new values on the flavonoid content of many foods in order to better determine the impact of flavonoid consumption against various chronic diseases [3,5]. Flavonoids, particularly flavan-3-ols, have been associated with a reduced risk of cardiovascular disease by modulating different primary and secondary prevention mechanisms [6]. Flavonoids are present in various sources in the plant kingdom and have a wide variety of biological properties. They have already proven their health benefits [3,7]. One of the most important activities is their role as antioxidants. As antioxidants, flavonoids are able to decrease oxidation of a substrate even in small amounts when compared to the substrate itself [8].

Food composition data describe food content in terms of nutrients and energy as well as non-nutrients such as phytochemicals, bioactive food components, anti-nutrients, or toxic compounds [9,10,11]. Food composition data are the basis of most nutritional studies [9]. Food sources are complex matrices in which antioxidant activity varies with the amount and type of bioactive compounds. The number of polyphenols in certain foods changes with different factors. For example, the phenolic composition of fruits varies widely among cultivars [12]. Therefore, the antioxidant capacity of food is itself variable [13,14].

Several methods are available for determining the type and amount of antioxidants in the diet. Prior studies (2015) described that, depending on the reactions involved, these assays can be classified into two types: hydrogen atom transfer reaction (HAT) based assays and electron transfer (ET) based assays. Among them, the oxygen radical absorption capacity (ORAC, classified as HAT) [1] has emerged as a test of choice in measuring the peroxyl radical scavenging capacity in foods and other matrices [15]. The correct use of the data obtained by this methodology through epidemiological clinical trials has broadened the knowledge about the dietary intake of antioxidants and their relationship with chronic diseases [16,17,18,19,20,21,22,23]. These epidemiological studies support the concept that food intake of ORAC (Oxygen Radical Absorbance Capacity) compounds above 10,000 µmol TE (Trolox equivalents) is related to a decreased risk or incidence of hypertension and cerebral infarction [1]. However, the data available in the scientific literature on ORAC for food cannot yet cover a wide range of examples and are limited to the associated eating habits in very specific regions.

The present study takes into consideration the need for a holistic and less reductive treatment in the analysis of health benefits of bioactive compounds in the nutritional sciences [24]. Our goal is to consider the analysis of the FCDB from a chemo-informatic perspective, which aims to generate useful models that can predict the chemical and biological properties of compounds [25]. Essentially, this research is based on the assumption that the countless data from the FCDB have enormous chemical information due to the structural diversity of the compounds encoded therein. Several articles have explained that antioxidant properties are also related to the chemical structure of polyphenols, and mainly attributed to the high reactivity of hydroxyl substituents [26,27]. 

Although the study is focused on flavonoid content data, it is necessary to recognize the existence of proanthocyanidin (PA) FCDB [7]. This database was created based on the growing number of studies that reveal health benefits associated with ingestion of PA, *per se* or in conjunction with other flavonoids [7,28,29,30]. As an example, procyanidin may be highlighted. Its oligomeric state has been shown to contribute to the antioxidant activity of various matrices [31,32,33]. These complex structures cannot be coded correctly by the chemoinformatic software used in this project. Therefore, we decided to start the study with the monomeric flavonoids included in the USA National Nutrient Database (USDA) and in a different FCDB.

This project was developed considering the possibility of generating predictive information related to the data found in the FCDB. We were looking for a tool to predict the antioxidant capacity of foods containing different compounds with flavonoid scaffolds (exogenous antioxidants in the diet). As stated earlier, data on food composition are complex and extensive [34]. Therefore, it is difficult to process all the information regarding the different essays presented in the bibliographic sources. Information processing is still performed by classical statistical methodologies [35,36]. However, when the problem is complex and mediated by nonlinear behaviors, it can be studied from a multivariate perspective or using artificial intelligence (AI) techniques [37,38]. In the biomedical field, several unidirectional supervised networks were used, especially based on the MultiLayer Perceptron (MLP). In chemoinformatic studies, researchers used other methods of Machine Learning ML [39]. In the nutrition sciences, the need to use ML models for personalized nutrition has recently been raised [40]. However, as far as we know, these techniques have never been used for the analysis and study of the FCDB. Therefore, current work is focused on obtaining optimal models based on ML methods that allow for predicting the total antioxidant capacity of foods, based on information from the flavonoid composition database and structural topological descriptors of flavonoids.

## 2. Materials and Methods 

### 2.1. Conformation of the Data Related to the Food Composition

Information from the dataset was obtained from different FCDB: (a) database for the flavonoid content of selected foods, version 3.1 and (b) isoflavone database released by the USDA in 2008 [3,5]. Therefore, estimation techniques were used to calculate unavailable values and the decision-making procedure described by Bhagwat et al. (2015) [35]. This information was used to prepare the dataset related to the composition of flavonoids in different foods. The standard reference (SR) was used to identify very unique food intake [7].

### 2.2. Prediction Using ML Algorithms

The prediction followed two phases, with different purposes: (i) selection of the attributes that best relate to the class (set A1). Metaheuristic Particle Swarm Optimization + Rougt Set Theory (PSO + RST) techniques were used [41,42], which included obtaining optimal prediction models among the selected ML algorithms using the hierarchical attributes of set A2 and their validation. To facilitate the experimentation of ML algorithms and the optimization capacity, the R language was used. This language also allowed the creation of each of the models corresponding to the three ML algorithms for predicting the antioxidant capacity. The interpolation package train function (Classification and Regression training) was used to evaluate the ML algorithms using the same metric and validation techniques.

Description of the Class Variable. The selected variable (attribute class) to predict was the ORAC value (ORACexp) was expressed in µmol TE/100g. ORAC was selected because it is considered the preferable methodology to evaluate antioxidant capacity. This is due to its correlation with antioxidant efficacy in vivo [43]. This assay was used to measure the antioxidant activity of foods. The assay measures the degree of inhibition of peroxyl radical induced oxidation by the compounds of interest in a chemical medium. The analytical method developed by Prior et al. (2003) was used as a reference method for selected sources [44].

Training Set and Test Set. As an internal validation methodology, the k-fold cross-validation method of k = 10 iterations was used for all algorithms [3].

#### 2.2.1. Selection of Attributes

Attributes Selection. For the attributes, different weights were assigned considering their influence on the attribute class. The attributes (set A1) were:

1. Flavonoid value equivalent to the antioxidant capacity of Trolox (TEACexp),
2. Flavonoid class (Class_flav),
3. Flavonoids (id_flav),
4. Amount of flavonoids (mean_flav),
5. Total value of polyphenols (TPexp),
6. Structural-topological characteristics (spectral moments, μk^w^, where ^w^ is bonding weights)

The experimental parameters were taken from the available scientific literature. TPexp (GAE mg/100 g) was found for each substrate.

The structural-topological attributes used for the study were the molecular descriptors (μk) of the Topological Sub-structural Molecular Design (TOPSMODE) approach [45]. The spectral moments of each flavonoid were calculated from their Simplified Molecular Input Line Entry Specification (SMILES) using MODESLAB software (version 1.0) and weighted for different binding properties. These bonding weights used in the present work describe the n-octanol/water partition coefficient (H), polar surface (PS), polarizability (Pol), Gasteiger-Marsilli charge (Ch), van der Waals atomic radii (vdW), and molar refraction (RM). An extensive dataset was created with the structural-topological information of flavonoids present in foods.

Attributes Hierarchy. The following relationships were analyzed: (i) the relationship between the attributes of set A1 and the class variable was investigated, and (ii) the influence of new attributes related to the structural-topological information of flavonoids in the class was evaluated. The working hypothesis was based on the existence of a relationship between the chemical structure of each flavonoid and the total antioxidant activity of the studied food matrices.

To select the attributes (A2), a ranking ranked according to their relationship with the class was formed. Different weights were assigned to each attribute using the quality measure of a similarity decision system. Weights were assigned manually and using PSO + RST, implemented in Java.

#### 2.2.2. Obtaining and Validating the Optimal ML Models 

To develop the training process, the *caret* package (classification and regression training) was used through the RStudio version 0.99.441 tool. This allowed the R language to be used in all experiments.

For data preparation, the database contained in a .csv file was imported. The data was divided into a training dataset with 75% of the inputs and the remainder with 25% using the *createDataPartition ()* function (*createDataPartition* (totalData $ total.orac, *p* = 0.75, list = FALSE)).

Attribute set A2 was selected for this study. The *in silico* influence of each attribute was considered in the class variable, which results from phase 1. In this phase, four algorithms were implemented:

(a) nearest k-neighbor algorithm (KNN) (where the optimized parameter was the integer, such that k € [1,10].
(b) The Support Vector Machine (SVM) algorithm required the use of the *kernlab* package and the radial base function of the *kernel* function, which allows the optimization of sigma parameters according to C (evaluated in an incremental range from smallest to highest).
(c) The MLP algorithm was used optimizing the size parameter, which represents the network size given by the number of internal layers it has. The values were assigned over a wide range to evaluate the trend following the best predictions and, thus, select the appropriate number for the parameter. The defined vector (c (1,4,3,5,7,9,10,11,12,15,20,25,50)) was performed using TuneGrid function.
(d) In the Random Forest (RF) algorithm, *mtry* and *ntree* parameters were defined. The optimal value in this case was 3. For a more comprehensive experiment, it was considered that the use of *ntree* is generally treated with values of 500 or more, depending on the data and vectors *seq* (3,4,5,6) and *seq* (500,600,700) for *mtry* and *ntree*, respectively.

The resulting optimal models were validated using test suites. The *predict* function was used. It was found that the models chosen were not adjusted and the best performance model was established. For this, graphical functions and calculation of the metrics present in the R language were used.

Experiment 1: Comparison of the outputs of the KNN, SVM, RF, and MLP algorithms generated in training with those generated in predicting the test suite. The goal is to determine the excess of fit in the models and which of the performances is the best. This was done through the *plotObsVsPred* function belonging to the interpolation package. A graph with the content of the reticular diagrams of each model was generated in the training and test sets. Model error metrics were calculated in the test phase using *mmetrics* from the *rminer* package. The parameters were two numerical vectors that represent the original outputs of each instance and the predicted outputs.

Experiment 2: Comparison of predictions for new values of total antioxidant capacity in each model. The objective is to determine the accuracy of the antioxidant capacity predictions corresponding to the new compounds, by comparing them with the original ones, and by characterizing the best predicted occurrences. A *dataframe* was used, containing the output values of each algorithm and those of the original set, generated by the *extractPrediction* function of the interleaving package. The graphs were generated with the prediction values and their originals by instances, which were represented in a Cartesian coordinate system.

## 3. Results and Discussion

This project focused on the idea that dietary antioxidants are substances that significantly decrease the adverse effects of reactive species, such as reactive oxygen and nitrogen species, among normal physiological functions in humans [46,47]. Due to the complexity of food composition, it is not completely known which diet constituents are responsible for health benefits, but antioxidants appear to play an important role [48,49].

### 3.1. Database Description

**Figure 1:**
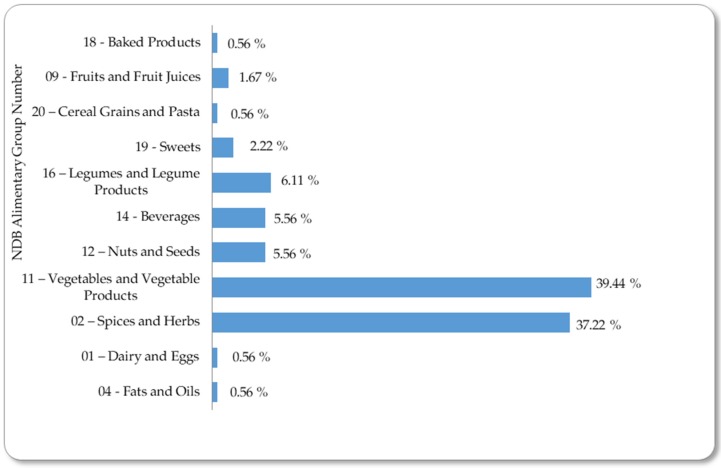
Percentage of each NDB (Nutrient Database) alimentary group represented in the studied dataset.

The database used to create the templates consisted of 991 entries, six different types of attributes, and the class. Therefore, the resulting matrix has a high dimensionality. The studied feeding matrices were divided into 11 groups according to NDB (Nutrient Database) Alimentary Group Number [3]. Vegetables, spices, and herbal herbs are the two groups with the most flavonoid-containing foods, accounting for 39% and 37%, respectively (Figure 1). In this dataset, high variability in flavonoid content predominated. This has been similar for all dietary polyphenols [50]. Several factors that affect the content of polyphenols in foods have been described [51,52].

The monomeric food flavonoids present in the data studied (id_flav attribute) belong to the chemical subclasses: flavonols, flavones, flavanones, and flavan-3-ols (Table 1). Quantifying them as aglycones facilitated the analysis but reduced the variety of compounds that could be analyzed. Flavonoids of the anthocyanin subclass can be found in many foods. Total anthocyanidin content in plant sources and extracts was correlated with the ORAC values. Anthocyanins constitute one of the most studied subclasses in the field [53]. Food intake of anthocyanins is high compared to other flavonoids due to their wide distribution in plant materials [54]. However, they were not included in this study because of their structure, which invalidates the application of the TOPSMODE approach [45].

Chemical structures, SMILE codes, and some examples of sources of the studied flavonoids are shown in Table 2.

### 3.2. Hierarchy Analysis of Attributes

Table 3 shows the order of influence of the attributes on the predictor variable (class). This order is associated with a higher "weight" in qualifying for this data matrix (dataset). Total polyphenols is the most important factor in predicting the total antioxidant capacity of foods. Although no history of this correlation is reported by AI algorithms, there are reports in which linear correlation was observed for more limited datasets. For example, positive correlations between ORAC and total phenolic content have also been previously reported [59].

In addition, the introduction of structural-topological information as new metadata helped to verify the hypothesis that the chemical structure of the food flavonoids is correlated with the total antioxidant capacity. The influence of these topological weights or structural attributes is limited to this database. However, the high dimensionality of the matrix and the fact that the food is compiled in the FCDB led to the suggestion that the scope of these results is correlated with the knowledge currently available in this field.

The molecular descriptors that most influence the class are presented in Table 3. All molecular descriptors (Table 3) are referred to as the n-octanol/water partition coefficient. For this reason, in the data series analyzed, this link property is the one with the most influence. The hydrophobicity of flavonoid diphenylpyran scaffolding may also influence antioxidant capacity [60]. The improved ORAC test provided a direct measure of hydrophilic and lipophilic antioxidant breaking ability in the presence of peroxyl radicals [61,62].

The amount of each flavonoid in the food matrix exert less influence (0.0341), as well as the antioxidant activity of the flavonoid compounds, especially TEACexp (0.0109). This may be related to the fact that antioxidant levels in foods do not necessarily reflect their total antioxidant capacity, which also depend on the synergistic and redox interactions between different molecules present in foods, which are not included in the dataset studied [48].

### 3.3. Models Obtainment and Validation

#### 3.3.1. Training Model

For the KNN algorithm and an optimal value for the k = 1 training model, the metrics produce the best results (small RMSE, Root Mean Squared Error) (Table 4). These results are superior to the models obtained in previous studies (RMSE = 5,475,398) [63]. This may be due to the features offered in the R language, which beneficially contribute to the model validation process and parameter optimization, as well as avoid excessive adjustments. It was also important to include structural-topological information as a highly influential attribute in the variable class.

For experimentation with RF, parameters such as *mtry* and *ntree* were defined. The optimal value (for regression problems) is known to be given by the third part of the number of descriptors for *mtry* (in this case, it would be 3). For the *ntree*, it is common to be treated with values of 500 or more, depending on the date. The vectors *seq* (3,4,5,6) and *seq* (500,600,700) were defined for *mtry* and *ntree*, respectively, in order to make the experimentation a little more comprehensive. The optimal model was obtained with the values of *mtry* = 6 and *ntree* = 500.

The MLP neural network was used for model adjustment. In this case, the size parameter has been optimized, which represents the network size provided by the number of inner layers. The values were assigned over a wide range to evaluate the trend by following the best predictions and, thus, selecting the appropriate number for the parameter. Therefore, the vector c (1,4,3,5,7,9,10,11,12,15,20,25,50) is defined through *tuneGrid*. From the resulting models, the best predictor was obtained by applying the size parameter with the value 4, even though its performance was lower than in other experiments.

Regarding the analysis performed with the SVM algorithm, the results of the vector were obtained for the values of sigma c (0.03,0.30,3.30,36,3,399,30) and distribution C (1,10,16,32,64,128,256,512.1024). The statistics for the Radial Basis Function core function experiment were: Sigma (σ) (399.3), C (10), RMSE (1853.446), Rsquared (0.879), RMSE SD (1370.442), and Rsquared SD (0.166). Subsequent analysis of the intervals around the σ and C values led to the definition of a new lower limit for the vector calculation. The optimal value was found for SVM (Table 4). This value was obtained for the new vector of σ and was c (1,11,121,1331). In this case, the optimal model was reached with σ = 121 and C = 10.

#### 3.3.2. External Validation

Validation of optimal models was performed using the test sets. For this, the prediction function was used as a parameter. Error metrics for the results of each model (Table 5) allowed us to indicate the RF algorithm as the best performance in this validation phase, determined by RMSE and R^2^ errors.

**Figure 2:**
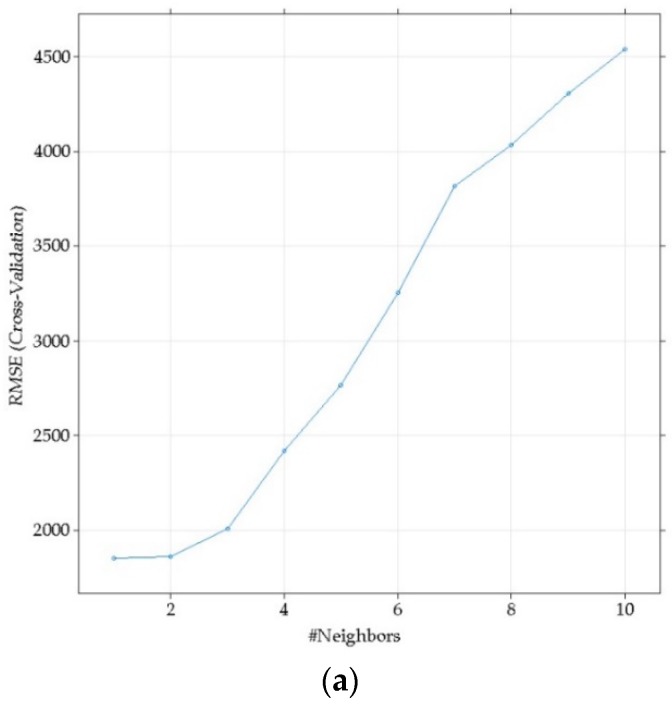

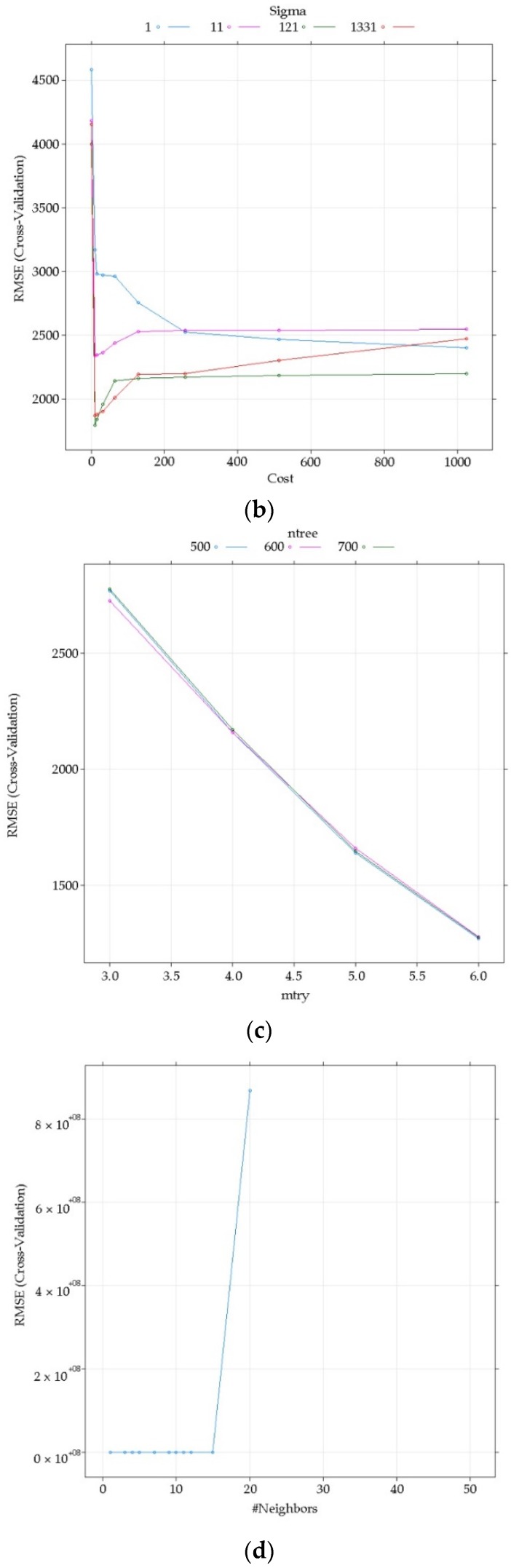
Effectiveness performance versus RMSE (Root Mean Squared Error) for each algorithm. (**a**) KNN (nearest k-neighbor algorithm). (**b**) SVM (Support Vector Machine). (**c**) RF (Random Forest). (**d**) MLP (Multi-Layer Perceptron).

The performance of the RMSE metrics for each of the algorithms in the parameter optimization process is shown. For the KNN algorithm (Figure 2a), as the parameter k increases number of neighbors (#Neighbors), the greater the error becomes. The results for SVM are shown in Figure 2b, where each row represents a value σ, distributed according to Cost (C) across the X axis. In this case, σ = 121 for C = 10, the optimal parameters are shown. Figure 2c corresponds to the RF algorithm. Each line represents the number of trees generated by the algorithm in each case (*ntree*). Points are models with the corresponding *mtry* value. The error tends to decrease as you approach a higher level for MLP. Error behavior is observed by varying the size parameter, which tends to increase abruptly from size = 15.

#### 3.3.3. Effectiveness Performance Comparison 

Experiment 1. Model prediction results for metrics in the training and testing phases are shown in Table 4 and Table 5. In all cases, the superiority of the model corresponds to RF, which is followed by SVM and KNN. In the case of the MLP neural network, a very poor performance at both times was recorded.

**Figure 3:**
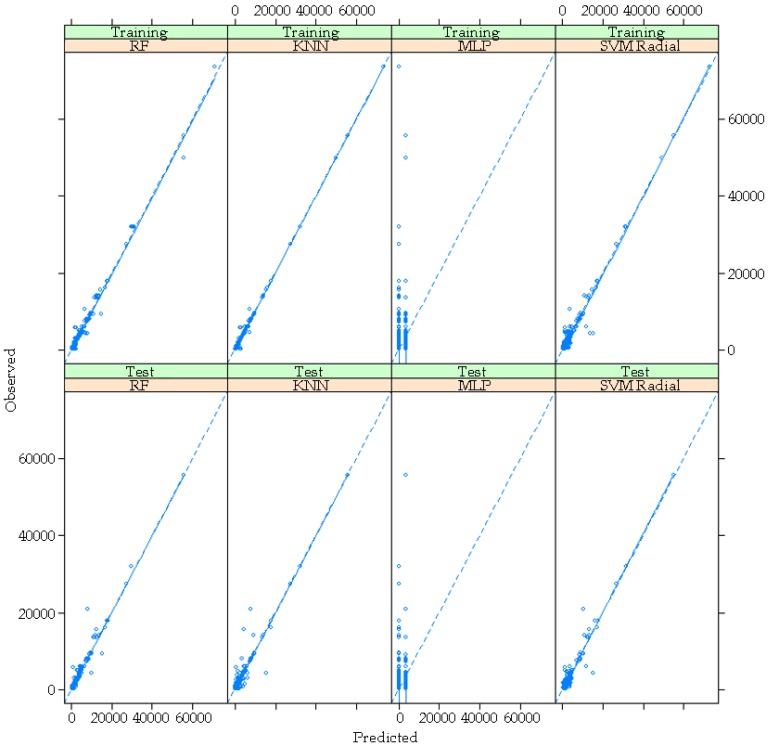
Representation of the numerical outputs in each of the models for the training and tested dataset.

Predictions have adequate accuracy and low over-fit rate, except for the MLP model (Figure 3). A comparison between the training moment and the test moment in each model shows similarity in the distribution of the output values around the reference line.

**Figure 4:**
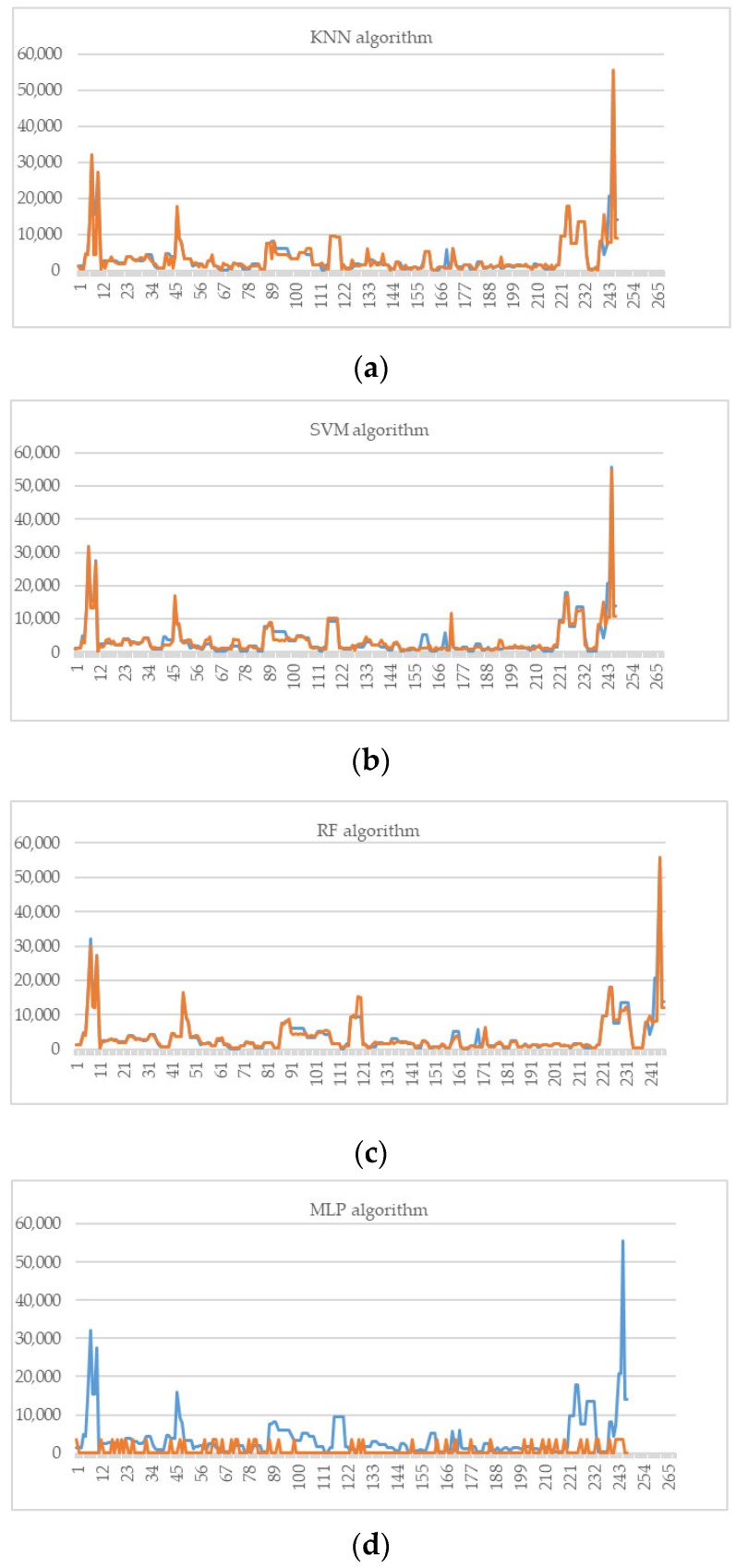
Representation of numerical outputs in each model for training and dataset tested (**a**) KNN, (**b**) SVM, (**c**) RF, and (**d**) MLP.

**Experiment 2.** The models corresponding to the SVM, KNN, and RF algorithms show an accurate prediction of new instances. The lines representing the vectors of the original and predicted values have a similar path except for the MLP model (Figure 4).

The optimal models obtained demonstrate the good effectiveness that can be achieved using AI algorithms. Only a small set of foods belonging to a specific food group or type was studied. An important and innovative feature of the present study is the size of the matrix, which represents the very large data set and describes various food groups. Prediction of the antioxidant capacity of foods by the ORAC method has not been documented, which makes it difficult to compare different methodologies. In the field of food, the use of data mining techniques is, therefore, untapped. However, there are recent studies that use traditional regression methods to predict a specific antioxidant property [64,65,66,67,68,69].

The complex role of diet in chronic diseases is difficult to understand, since a typical diet provides large amounts and different types of bioactive components. These bioactive molecules can modify a multitude of processes related to these diseases. Due to the complexity of this relationship, a comprehensive understanding of the role of these bioactive components is required in order to assess the role of food in modulating human health and disease. Food composition data alone does not provide this knowledge. However, processing your data and information obtained may be useful for further studies and to complement in vivo and ex vivo studies. Based on the current study, the total antioxidant capacity of foods can be predicted whenever their TPexp and the structural-topological information of the flavonoids they contain are known. The obtained models were automated in a software (PCAT, version 1.0), whose functionalities allow the validation of each model with a new data set and, therefore, new predictions.

## 4. Conclusions

The *in silico* methodology developed allows us to confirm the effectiveness of the models obtained through the introduction of the new structural-topological attributes, as well as the selection of those that most influence the class variable, determined by the calculation of the PSO + RST algorithm. The RF algorithm shows the best quality parameters, both in the training and validation phases, which are the most successful. It is worth mentioning the use of R as the language and work environment, which allows the optimization of the algorithms’ parameters that led to the results. These predictions are limited to the FCDB and its metadata. There are new possibilities for learning ML models from new datasets, which is facilitated by their implementation in an automated predictive system in the development phase. The practical utility of the research is directed toward the generation of predictive theoretical knowledge, which is useful in the development of regional or local FCDB, dietary interventions, new nutritional studies, etc. It is an important antecedent in the “omics” disciplines applied to food and nutrition sciences, which lead to the analysis of a complex data system to obtain information using bioinformatic tools.

## Figures and Tables

**Table 1:** Examples of the conformation of the dataset and the respective attributes.

(NDB No)-ALIMENTARY GROUP ^a^	FOOD ^a^/NDB No.	ATTRIBUTES					CLASS (ORAC _EXP_) Mean
		Flavonoid ^a^	Class of Flavonoid ^a^	Amount of Flavonoid (Mean) ^a^	TEACexp ^b^	TPexp Mean	
(11)—Vegetables and Vegetable Products	Broccoli, raw (Brassica oleracea var. italica)/11090	(+)-Catechin	Flavan-3-ols	0	2.4	316 ^c^	1510 [13,14,55,56]
		(-)-Epigallocatechin 3-gallate	Flavan-3-ols	0	4.93		
		Hesperetin	Flavanones	0	1.37		
		Naringenin	Flavanones	0	1.53		
		Apigenin	Flavones	0	1.45		
		Luteolin	Flavones	0.8	2.09		
		Kaempferol	Flavonols	7.84	1.34		
		Myricetin	Flavonols	0.06	3.1		
		Quercetin	Flavonols	3.26	4.7		
(02)—Spices and Herbs	Guava, red-fleshed/99428	Apigenin	Flavones	0	1.45	247 ^d^	1990 [57]
		Luteolin	Flavones	0.8	2.09		
		Kaempferol	Flavonols	0	1.34		
		Myricetin	Flavonols	0	3.1		
		Quercetin	Flavonols	1	4.7		

^a^ Extracted from FCDB [3,5]. ^b^ Extracted from [58]. ^c^ Extracted from [14]. ^d^ Extracted from [57]. Trolox equivalent antioxidant capacity flavonoid value (TEACexp). Total polyphenol value (TPexp). Nutrient Database Number (NDB No).

**Table 2:** Examples of the chemical information of flavonoids, and their presence in food, contained in the studied database.

FLAVONOIDS	STRUCTURE	SMILE	NAME FOOD	NDB No. ^a^
(-)-Epicatechin 3-gallate	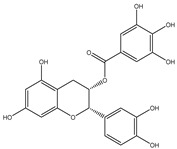	C1C(C(OC2=CC(=CC(=C21)O)O)C3=CC(=C(C=C3)O)O)OC(=O)C4=CC(=C(C(=C4)O)O)O	Apples, Fuji, raw, with skin	09504
(+)-Catechin	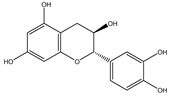	OC1CC2=C(O)C=C(O)C=C2OC1C3=CC=C(O)C(=C3)O	Bananas, raw (*Musa acuminata* Colla)	09040
Hesperetin	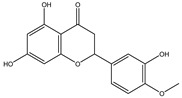	O=C(CC(C3=CC(O)=C(OC)C=C3)O2)C1=C2C=C(O)C=C1O	Juice, orange, raw	09206
Naringenin	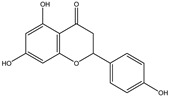	OC1=CC=C(C=C1)C2CC(=O)C3=C(O2)C=C(O)C=C3O	Melons, honeydew, raw (*Cucumis melo*)	09184
Apigenin	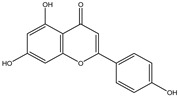	O=C(C=C(C3=CC=C(O)C=C3)O2)C1=C2C=C(O)C=C1O	Pineapple, raw, all varieties (*Ananas comosus*)	09266
Luteolin	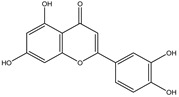	O=C(C=C(C3=CC(O)=C(O)C=C3)O2)C1=C2C=C(O)C=C1O	Pomegranates, raw (*Punica granatum*)	09286
Kaempferol	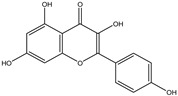	O=C(C(O)=C(C3=CC=C(O)C=C3)O2)C1=C2C=C(O)C=C1O	Broccoli, cooked, boiled, drained, without salt	11091
Quercetin	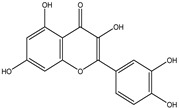	O=C(C(O)=C(C3=CC(O)=C(O)C=C3)O2)C1=C2C=C(O)C=C1O	Mushrooms, white, raw (*Agaricus bisporus*)	11260
Myricetin	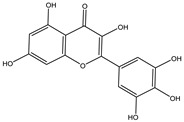	O=C(C(O)=C(C3=CC(O)=C(O)C(O)=C3)O2)C1=C2C=C(O)C=C1O	Potatoes, red, flesh and skin, raw (*Solanum tuberosum*)	11355

^a^ Nutrient Database Number (NDB No) [3].

**Table 3:** Hierarchy of attributes of the set A1 regarding their influence in the class.

Order	Attributes	Correlation Value ^f^	Set of Attributes for the Model
1	TPexp ^a^	0.1551576	A2
2	µ8 ^H^	0.1483031	A2
3	µ12 ^H^	0.1349679	A2
4	µ11 ^H^	0.1213032	A2
5	µ10 ^H^	0.1206462	A2
6	µ13 ^H^	0.1096691	A2
7	id_flav ^b^	0.1018874	(-)
8	mean_flav ^c^	0.0341301	(-)
9	TEACexp ^d^	0.0108586	(-)
10	Class_flav ^e^	0.0094634	(-)

^a^ TPexp (Total polyphenol value). ^b^ id_flav (Flavonoids). ^c^ mean_flav (Amount of flavonoid (mean). ^d^ TEACexp (Trolox equivalent antioxidant capacity flavonoid value). ^e^ Class_flav (Class of flavonoid). ^f^ Value of correlation with the class. (-) not selected for the model. ^H^ bonding weight n-octanol/water partition coefficient.

**Table 4:** Statistics corresponding to the training set score for the optimal models of each of the ML algorithms.

Algorithm	RMSE	Rsquared
KNN	1851.174	0.905
RF	1271.060	0.957
MLP	6582.955	0.284
SVM	1790.536	0.901

ML: Machine Learning; RMSE, Root Mean Squared Error; KNN: nearest k-neighbor algorithm; RF: Random Forest; MLP: Multi-Layer Perceptron; SVM: Support Vector Machine.

**Table 5:** Statistics corresponding to the test set score for the optimal models of each of the ML algorithms.

Algorithm	RMSE	Rsquared
KNN	1956.810	0.880
SVM	1622.627	0.917
RF	1557.108	0.925
MLP	6429.185	0.007
